# Prolonged cognitive–motor impairments in children and adolescents with a history of concussion

**DOI:** 10.2217/cnc-2016-0001

**Published:** 2016-05-12

**Authors:** Marc Dalecki, David Albines, Alison Macpherson, Lauren E Sergio

**Affiliations:** 1 School of Kinesiology & Health Science, York University, Toronto, Ontario, Canada; 2 Centre for Vision Research, York University, Toronto, Ontario, Canada; 3 York University Sport Medicine Team, York University, Toronto, Ontario, Canada

**Keywords:** cognitive–motor integration, performance, youth

## Abstract

**Aim:**

We investigated whether children and adolescents with concussion history show cognitive–motor integration (CMI) deficits.

**Method:**

Asymptomatic children and adolescents with concussion history (n = 50; mean 12.84 years) and no history (n = 49; mean: 11.63 years) slid a cursor to targets using their finger on a dual-touch-screen laptop; target location and motor action were not aligned in the CMI task.

**Results:**

Children and adolescents with concussion history showed prolonged CMI deficits, in that their performance did not match that of no history controls until nearly 2 years postevent.

**Conclusion:**

These CMI deficits may be due to disruptions in fronto-parietal networks, contributing to an increased vulnerability to further injury. Current return-to-play assessments that do not test CMI may not fully capture functional abilities postconcussion.

Concussion, also known as mild traumatic brain injury, can sometimes leave its trace on an individual's life by hindering cognitive and physical function for years afterward. For young children and adolescents aged 0–19 years, the estimated incidence of concussions is reported to be about 175,000 individuals treated annually in the USA for nonfatal sport-related concussion [1]. In addition, a principal feature of concussion is that once one had a concussion, it is easier to get another one [2,3]. In particular, it seems that children and adolescents are somewhat more vulnerable to concussion compared with young adults [3]. It has been suggested that this vulnerability is due in part to less developed cranial bones and neck musculature, a larger head-to-body ratio, and a higher amount of subarachnoid space, leaving more room for the brain to move within the skull [4]. In terms of repetitive head injury, behavioral studies have found an up to fivefold increase in mild cognitive impairment (MCI) and earlier onset of Alzheimer's disease (AD) among retired contact-sport athletes [5]. Moreover, reports have noted short-term memory loss, headache and migraine suffered 10–20 years following sport-related concussion [6]. Hence, there is a strong need to fully understand the mechanism(s) that underlie this increased vulnerability in young athletes, given the potentially dire consequences of multiple concussions [7].

There exist several assessment tools, including balance (e.g., leg stand tests, force plate tests), vestibular, oculomotor, symptoms and neuropsychological tests (e.g., ImPact, CogSport, SCAT3), which are used in present return-to-play protocols aiming to protect children and adolescents from risk of reinjury [6,8–13]. There remains a lack of robust metrics for clinicians and coaches to thoroughly assess relevant on-field function following concussion prior to safe resumption of preinjury activities, since it is increasingly clear that motor and/or cognitive deficits remain for longer than a few weeks and/or the return to play [10]. Further evidence that the current metrics may not fully capture recovery comes from a recent study demonstrating how brain activity of young adults did not return to preinjury status even after 12 months, despite being asymptomatic after 7 days and cleared for sport participation after 10 days [14]. Similarly, recent neuroimaging studies show evidence that despite a return to premorbid status (based upon current clinical measures), there are still residual deficits within brain structural and functional networks in the subacute phase of concussion [15–18]. Lastly, a recent study of over 700 varsity athletes found that almost half of the athletes who reported being symptom-free nevertheless failed to pass their neurocognitive test for a further 3 days, on average [19]. Hence, there may be a disconnection between what is being reported by the athlete and quantitative measures used to assess function. Such a delay has the potential for serious consequences since an athlete typically returns to full play immediately after being fully cleared and has passed all steps of the return to play protocols, perhaps before they are fully recovered functionally.

One possible explanation for these disconcerting findings might be that the current return-to-play protocols (compare section above) all test motor skills and cognition separately. During many tasks in sport (and in daily life), one has to act and think simultaneously. For example, when a hockey player skates to the left while stickhandling the puck around obstacles, he/she must also covertly attend to other players on the ice while considering where on net to shoot the puck. Sometimes this type of task, known as cognitive–motor integration (CMI), also requires a decoupling of vision and action [20,21]. In contrast, direct object interaction (the motor skill typically tested) does not require a decoupling of vision and action, for example, when you reach for an apple and grab it. Our group has previously shown that young adults with a history of concussion, although asymptomatic and returned to full play, show performance deficits during a CMI task and when there is dissociation between vision and action [22]. We have also observed similar performance deficits in older adults at risk for or in the early stages of AD [23,24]. These deficits are related to underlying changes in the fronto-parietal networks necessary for the successful integration of thought and action in collaboration with other brain areas, such as the cerebellum [25–28]. Notably, testing retired football and hockey players following a concussion history in their career showed significant impairments in the diffusivity in fronto-parietal networks [29] which are areas found to be crucial to properly perform rule-based movement [21,28,30–32].

Another issue that arises in relation to feasible assessment tools is that they should provide a simple and quick assessment in clinics and field settings. This is currently not the case with settings that use EEG or brain imaging techniques that are more appropriate for research purposes [33,34]. The main aim of the present study was, therefore, to investigate performance decrements of children and adolescents with a history of concussion when performing a feasible, side-line accessible CMI task, compared with healthy age-matched controls without concussion history. Based on our previous findings with young adults [22], we hypothesize that children and adolescents with concussion history will show impairments in their CMI performance, especially when there is dissociation between vision and action requiring rule-integration for successful performance. We predict these decrements will show up despite intact basic movement and cognitive performance (i.e., asymptomatic by current standards). We also hypothesize that the effect on CMI performance by concussion is more pronounced in this youth sample relative to young adults, given their greater developmental vulnerability. A second aim was to quantify the recovery of cognitive–motor performance in this population following concussion. This aspect of our study was more exploratory in nature.

## Material & methods

### Subjects

Fifty children and adolescents with a history of sport-related concussion (12.84 ± 1.61 years; 25 females) and 49 healthy aged matched controls (11.63 ± 1.93 years; 17 females) participated in this study. For the concussion history participants, the last concussion was on average 12.87 ± 10.42 months prior to testing (range: 0.25–48 months). We obtained the information about the concussion history and demographic data from all participants by our own and established questionnaires (SCAT3, Child-SCAT3). We interviewed the participant, the parents, and the coaches, in order to obtain as precise a self-reported concussion history as possible. The detailed listing of concussion history for each participant is summarized in Table 1. At the time of testing, all participants were reported to be healthy and were not diagnosed with concussion, and participants with concussion history were defined as ‘asymptomatic’ in accordance with current return-to-play protocol guidelines [10,35], and were fully participating in their team sport. Concussion history and control participants were recruited from the same hockey and soccer teams. The study protocol was approved by the Human Participants Review Sub-Committee, York University's Ethics Review Board, and conformed to the standards of the Canadian Tri-Council Research Ethics guidelines. Parents and children/adolescents signed a written informed consent/assent forms before participating in this study.

**Table 1.** **Characteristics and concussion incidence for participants with concussion history and characteristics for participants with no history of concussion.**

**Concussion history**					**No history**		

**Sub.**	**Sex**	**Age (years)**	**Concussions (n)**	**Last concussion – time since (months)**	**Sub.**	**Sex**	**Age (years)**
1	F	12	1	0.5	1	F	14

2	F	12	1	16	2	F	14

3	F	13	1	24	3	F	13

4	F	13	1	40	4	F	14

5	F	13	1	N/A	5	F	13

6	F	13	1	25	6	F	13

7	M	12	1	2	7	F	14

8	M	12	1	9	8	M	9

9	M	12	1	13	9	M	9

10	M	10	2	14	10	M	9

11	M	14	2	10	11	M	10

12	M	13	1	12	12	M	10

13	M	13	1	24	13	F	12

14	M	9	1	6	14	F	12

15	M	14	4	12	15	F	10

16	F	15	1	4	16	F	12

17	F	13	1	N/A	17	F	11

18	F	14	1	0.25	18	M	11

19	F	14	1	10	19	M	12

20	M	13	4	16	20	M	12

21	F	15	1	0.5	21	F	11

22	F	13	1	2.5	22	M	13

23	F	14	1	12	23	F	11

24	M	12	1	7	24	F	11

25	M	11	1	16	25	F	15

26	M	11	1	13	26	F	15

27	F	11	1	9	27	M	10

28	F	16	2	7	28	M	11

29	M	11	1	2	29	M	12

30	F	16	1	7	30	M	12

31	M	12	1	27	31	M	13

32	M	12	1	N/A	32	M	13

33	M	11	1	0.5	33	M	13

34	F	14	1	N/A	34	M	13

35	M	13	1	19	35	M	13

36	F	11	1	30	36	M	12

37	M	13	2	27	37	M	13

38	F	15	2	22	38	M	13

39	F	17	1	6	39	M	13

40	M	12	1	18	40	M	12

41	M	13	1	N/A	41	M	12

42	M	13	3	12	42	M	13

43	M	13	1	48	43	M	13

44	F	11	2	0.75	44	M	8

45	F	14	1	18	45	M	8

46	F	10	1	12	46	M	9

47	F	13	1	7	47	M	8

48	F	15	1	10	48	M	8

49	M	13	1	4	49	M	8

50	M	13	2	7			

**Mean ± SD**		**12.84 ± 1.61**	**1.3 ± 0.71**	**12.84 ± 1.61**			**11.63 ± 1.93**

F: Female; M: Male; N/A: Not applicable; Sub.: Subject.

### Dependent measures

All participants of the present study participated in an eye-hand coordination task, where they had to slide a finger along touch screens from a home target to different peripheral targets in two different conditions. The dependent variables of interest were reaction time, movement time (MT), path length, peak velocity, as well as movement accuracy and precision. The different variables are described in detail in the following section.

#### Performance timing

Mean reaction time (RT) across trials was calculated as the time between disappearance of the central target and movement onset, that is, when the participant began the movement execution by sliding the cursor toward the target. MT was calculated as the time between movement onset and offset, thereby representing the ballistic initial movement without corrective movements. Total movement time (TMT) was calculated between movement onset and final movement end point (i.e., reaching the peripheral target). Corrective movement time (CMT) was calculated as the difference between TMT and MT, that is, the time the participants required for their movement correction until finishing their trial. Note that for any trial where the participant's first movement ended in the target, MT and TMT would be equal and CMT would be zero. The peak velocity (PeakVel) was the maximum velocity in ms during the ballistic movement.

#### Pathlength

Measurements of pathlength were recorded for each trial. The ballistic pathlength (BallPL) was quantified as the distance in mm between start and the first correction of the initial cursor movement (distance covered during the ballistic MT). Full pathlength (FullPL) was calculated as the distance between start and end location of the cursor movement. Movements comprised of curves or deviations from a straight path between the central and peripheral target would thus result in a longer pathlength. Corrective pathlength (CorrPL) was quantified by subtracting BallPL from FullPL.

#### End point analysis

Accuracy of the first ballistic movement was determined by computing the absolute on-axis (i.e., distance, AbsOn) and off-axis (i.e., direction, AbsOff) errors, which reflect components of reaching accuracy that have been shown to be controlled independently by the motor system [36]. The constant absolute error (AE) – that is, the end point accuracy – was determined as the distance between the average movement end point for each target location (∑ x/n, ∑ y/n) and the actual target position (defined by the x and y coordinates at the center of the target). Variable error (VE) – that is, the end point precision – was determined as the distance between the end points of the individual movements (σ2) from their mean movements.

#### Direction reversals

Direction reversals (DR) were only applicable in the horizontal reversal (HR) condition. They were calculated when there was a deviation of more than ±45° from the line in either direction between the center of the central and peripheral target during the first half of each movement, and were recorded as a percentage of completed trials.

### Procedures

**Figure 1.**
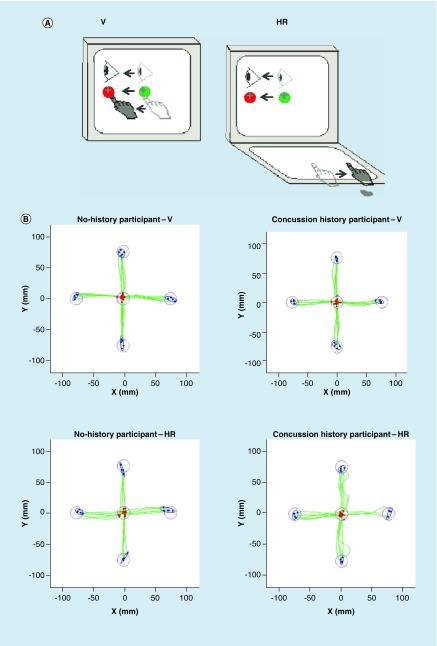
**Experimental setup and sample participant data.** **(A)** Schematic drawing of the both experimental conditions V and HR. Visual stimuli were presented on the vertical monitor for all conditions. Green circle, light gray eye and hand symbols denote the starting position for each trial (home target). Dark grey eye and hand symbols denote the instructed eye and hand movements for each task. Red circles denote the peripheral (reach) target, presented randomly in one of four locations (left, up, right and down). The dark crosshair denotes the cursor feedback provided during each condition. **(B)** Typical full hand path data of one participant with no history of concussion and one participant with concussion history, performing the V and HR condition. Note the poorer hand path in condition HR compared with V for both subjects, and the poorer hand path of the participant with concussion history compared with the participant with no history of concussion in condition HR. HR: Horizontal reversal; V: Vertical.

Participants performed two visuomotor transformation tasks, similar to those previously used for MCI and early AD (eAD) patients and young adult athletes with concussion history [22,24,37–39]. Participants sat on a chair at a desk where the experimental task was presented on an Acer Iconia 6120 dual-touch screen tablet, with a touch screen in the vertical and in the horizontal plane. The first condition (V, shortcut for ‘vertical’) was the standard mapping task, where the spatial location of the viewed target and the required movement (sliding a finger along the vertical touch screen to move a cursor from screen center to a peripheral target) was in alignment. The second condition (HR) was a nonstandard mapping task. This task required CMI with two levels of decoupling between vision and action: targets were again viewed on the vertical touch screen, but participants had to slide their finger – along the horizontal touch screen and in the opposite direction to displace the cursor (feedback reversal). That is, to move the cursor to the left, they had to slide their finger to the right, for example (compare Figure 1A).

In both conditions, trials were presented in a pseudo-randomized order. The peripheral targets presented on the vertical touch screen were of 20 mm diameter, red colored and presented to the left, right, above or below the central target (also 20 mm in diameter). The distance between the middles of the peripheral and central target (i.e., the screen center) was 75 mm. The task itself was displayed on a 170 × 170 mm black square with the surrounding background colored grey, in order to maintain a constant visual border. There were a total of 20 trials (i.e., five to each peripheral target) per condition, thus, altogether 40 test trials per participant. The order of performing the V and HR conditions was randomized across participants in each group. To ensure task comprehension, each participant performed two practice trials in each direction in both conditions.

The trial timing and responses of participants were as follows for the V condition: a green colored center target was presented on the vertical touch screen; participants touched the target with the index finger of their dominant hand – the target then changed its color to yellow; after holding the center target for 4000 ms, the red peripheral target appeared and the center target disappeared, serving as ‘go-signal’ for participants, that is, to concurrently look and slide their index finger along the touch screen to move the cursor to the target; once the peripheral target was reached and held for 500 ms, the peripheral target disappeared and the trial ended; the next trial began with the presentation of the center target after an intertrial interval of 2000 ms. The sequence was the same for the HR condition.

Participants were instructed to look at and move the cursor to the target as quickly and accurately as possible, and ambient distractions were kept to a minimum. Participants had full vision of their hand and fingers. Due to the possibility of cervical soft tissues injury following concussion and the need for portability, eye-tracking was not done. The experimenter monitored participant's eye movements during the experiment and if incorrect movements were made, participants were reminded to always look toward the target and not to their hand. Those incorrect movements were <2% of trials and were eliminated before final data analysis.

### Data processing

Response timing, finger position (x, y coordinates; 55 Hz sampling rate) and error data were recorded during each trial. Saved raw data were then converted into MATLAB readable format using a custom written C++ application. Individual movement paths derived from the cursor location were first low-pass Butterworth filtered at 10 Hz (filtfilt function, Matlab, Mathworks, Inc., Natick, MA, USA). Trials were deemed errors if the finger/cursor left the center target too early (<4000 ms), reaction time was too short (<150 ms), reaction time was too long (>8000 ms) or MT was too long (>10000 ms). Trials in which the first ballistic movement exited the boundaries of the center target in the wrong direction (i.e., moving the finger toward as oppose to away from the visual target >45° from a straight line to target) were coded as DR error and also eliminated from further analyses (but counted). Movement onset and ballistic movement end points (i.e., the initial muscle impulse before any movement path corrections) were scored using custom software as 10% PeakVel (c.f. [23]), then verified visually to ensure that computed offsets by the program appropriately reflected the first point at which movements slowed significantly. The total movement end point was then scored as the point at which velocity reached a final zero-crossing and position data plateaued (i.e., when subjects stopped the cursor inside the peripheral target). The processed data were then loaded into a custom written analysis program to compute different movement timing and execution outcome measures, described in detail below.

### Statistical analysis

For all dependent measures (RT, MT, TMT, CMT, PeakVel, BallPL, FullPL, CorrPL, AbsOn error, AbsOff error, AE and VE), effects of group (concussion history, no history) and condition (V, HR) were analyzed using a repeated-measures mixed ANOVA, with the between factor group (concussion history, no history) and the within factor condition (V, HR). When there were significant main or interaction effects, pair-wise comparisons were used. We additionally performed an ANCOVA with the covariates sex, age and number of concussions to check for potentially demographic or ‘concussion load’-related characteristics. Also, a separate analysis using sex and age as main effect was run to test for any sex-related or age-related behavioral differences. Percent DR's in HR were compared between groups using a one-way ANOVA. Trial whose value was >2 standard deviations away from the mean for a given condition in a given group was considered an outlier and removed before statistical analysis. All remaining data were checked for normal distribution (Shapiro–Wilk's test) and sphericity (Mauchly's test), and were Greenhouse-Geisser corrected where necessary. Statistical significance levels were set to 0.05, and all statistical analyses were performed using SPSS statistical software (IBM, Inc., NY, USA). Statistical significance levels were set to α = 0.05.

#### Level of dissociation

In order to test if performance declines would be significantly larger for the decoupled version of our task, that is, the nonstandard mapping condition (HR), we compared participants’ performance as a function of their change from standard mapping condition V (i.e., no decoupling between eye and hand) to HR. We subtracted out the result on the V condition from the HR condition for each given dependent measure, and performed a one-way ANOVA with the performance measure as dependent variable and the between factor group (concussion history, no history) for all dependent variables (RT, MT, TMT, CMT, PeakVel, FullPL, BallPL, CorrPL, AE, VE, AbsOn error and AbsOff error).

#### Relation between performance & concussion history

In order to investigate whether possible performance deficits were related to the time since the last concussion, we correlated (for the concussion history group) all dependent main variables (RT, MT, TMT, CMT, PL, BallPL, PeakVel, AE, VE, AbsOn, AbsOff and DR) with the time since last concussion (in months) with a linear and nonlinear regression analysis. Note that we had three participants (marked as ‘N/A’ in Table 1) without precise ‘time since injury’ data, which we excluded from the correlation analysis. Since these three participants fell into the time range of the other participants (-0.25–48 months postconcussion), we included them into the main analysis (compare above) to increase statistical power.

#### Discriminant analysis

In order to test if our task was sensitive enough to predict a presence of concussion history based on task performance between the no history and concussion history group, we also performed a separate step-wise discriminant analysis. The dependent measures that showed a statistical significant difference between concussion history and no history control participants were entered as predictor variables in a step-wise discriminant analysis comparing concussion history and control groups.

## Results

The analysis using sex and age as main effect yielded no significant differences for all dependent variables supporting the decision to merge data across sexes and age (all p > 0.05). Since it is well known that children's behavior and brain development can differ between the age range of 9 and 16 years [7,40], in a further analysis we subdivided the participant groups into two age groups (9–12 years and 13–16 years), and included both age groups as an independent between-factor variable in the ANOVA. This second ANOVA found no age-related performance differences (p > 0.05).

Examples of typical movement trajectories of one participant with concussion history and one control participant without concussion history performing the V and HR condition are presented in Figure 1B. Statistical outcomes of the repeated-measures mixed ANOVA for all dependent variables for group (concussion history, no history) and condition (V, HR) are summarized in Table 2 and descriptive statistics in Table 3.

**Table 2.** **Statistical outcome of the repeated-measures mixed ANOVA of group (concussion history, no-history) and condition (vertical, horizontal rotated) for all dependent variables and the one way ANOVA for direction reversal errors.**

**Variable**	**Group**	**Condition**	**Group × condition**
RT	F(1,97) = 0.19^†^	F(1, 97) = 232.55***	F(1,97) = 0.00^†^

MT	F(1,97) = 7.75**	F(1, 97) = 214.14***	F(1,97) = 4.93*

TMT	F(1,97) = 3.60^†^	F(1,97) = 352.84***	F(1,97) = 1.46^†^

CMT	F(1,97) = 0.08^†^	F(1, 97) = 238.66***	F(1,58) = 0.18^†^

PeakVel	F(1,97) = 1.13^†^	F(1,97) = 424.68***	F(1,97) = 0.98^†^

BallPL	F(1,97) = 18.11***	F(1,97) = 27.85***	F(1,97) = 6.04*

FullPL	F(1,97) = 11.03**	F(1,97) = 64.45***	F(1,97) = 6.20*

CorrPL	F(1,97) = 2.67^†^	F(1,97) = 153.42***	F(1,97) = 0.00^†^

AE	F(1,97) = 6.34**	F(1,97) = 109.15***	F(1,97) = 0.59^†^

VE	F(1,97) = 0.37^†^	F(1,97) = 0.65^†^	F(1,97) = 2.49^†^

AbsOn	F(1,97) = 11.50**	F(1,97) = 112.82***	F(1,97) = 1.89^†^

AbsOff	F(1,97) = 0.06^†^	F(1,97) = 80.87***	F(1,97) = 0.02^†^

**One-way ANOVA for DR in condition HR**			

DR		F(1,98) = 1.86^†^	

**Significant outcomes pair-wise comparisons**			

Parameter	Condition		

MT	HR	F(1,98) = 7.29**	

BallPL	V	F(1,98) = 9.38**	
	HR	F(1,98) = 15.57***	

FullPL	V	F(1,98) = 4.41*	
	HR	F(1,98) = 9.51**	

CorrPL	V	F(1,98) = 4.22*	

AE	V	F(1,98) = 7.24**	
	HR	F(1,98) = 5.22*	

AbsOn	V	F(1,98) = 7.52**	
	HR	F(1,98) = 8.78**	

^†^Non-significant values.

*p < 0.05.

**p < 0.01.

***p < 0.001.

AE: Constant absolute error, end point accuracy; AbsOff: Absolute off-axis error; AbsOn: Absolute on-axis error; BallPL: Ballistic pathlength; CMT: Corrective movement time; CorrPL: Corrective pathlength; DR: Direction reversal error; FullPL: Full pathlength; HR: Horizontal rotated; MT: Movement time; PeakVel: Peak velocity; RT: Reaction time; TMT: Total movement time; V: Vertical; VE: Variable error, end point precision.

**Table 3.** **Descriptive statistics of the repeated measures-mixed ANOVA of group (concussion history, no-history) and condition (V, HR) for all dependent variables and the one way ANOVA for direction reversal errors.**

**Variable**	**Condition**	**Concussion history (mean ± SD)**	**No history (mean ± SD)**
**ms**			

RT	V	495.77 ± 101.93	486.56 ± 104.03
	HR	687.72 ± 139.08	677.91 ± 146.54

MT	V	329.33 ± 122.52	290.04 ± 70.24
	HR	706.12 ± 269.70	567.54 ± 239.83

TMT	V	473.19 ± 199.60	417.07 ± 108.72
	HR	1153.66 ± 407.85	1015.38 ± 394.97

CMT	V	143.86 ± 104.70	127.03 ± 63.68
	HR	447.54 ± 243.68	447.85 ± 214.87

PeakVel	V	184.58 ± 46.76	188.64 ± 43.04
	HR	100.59 ± 33.72	112.35 ± 42.49

**mm**			

BallPL	V	65.61 ± 5.05	62.70 ± 4.40
	HR	63.34 ± 9.34	56.47 ± 7.94

FullPL	V	71.97 ± 2.67	70.86 ± 2.62
	HR	80.19 ± 10.47	75.18 ± 4.47

CorrPL	V	6.36 ± 4.18	8.16 ± 5.53
	HR	16.85 ± 8.95	18.72 ± 8.84

AE	V	9.51 ± 4.05	11.78 ± 4.32
	HR	16.29 ± 6.33	19.63 ± 8.11

VE	V	7.05 ± 2.50	8.03 ± 2.98
	HR	8.11 ± 3.34	7.68 ± 3.75

AbsOn	V	8.05 ± 4.59	10.65 ± 4.83
	HR	14.75 ± 6.62	19.33 ± 8.66

AbsOff	V	1.71 ± 0.80	1.67 ± 0.97
	HR	4.27 ± 3.44	4.15 ± 2.17

**Count (%)**			

**DR**	**HR**	**4.34 ± 0.52**	**3.45 ± 0.39**

AbsOff: Absolute off-axis error; AbsOn: Absolute on-axis error; AE: Constant absolute error, end point accuracy; BallPL: Ballistic pathlength; CMT: Corrective movement time; CorrPL: Corrective pathlength; DR: Direction reversal errors; FullPL: Full pathlength; HR: Horizontal rotated; MT: Movement time; PeakVel: Peak Velocity; RT: Reaction time; SD: Standard deviation; TMT: Total movement time; V: Vertical; VE: Variable error, end point precision.

### Performance timing

**Figure 2.**
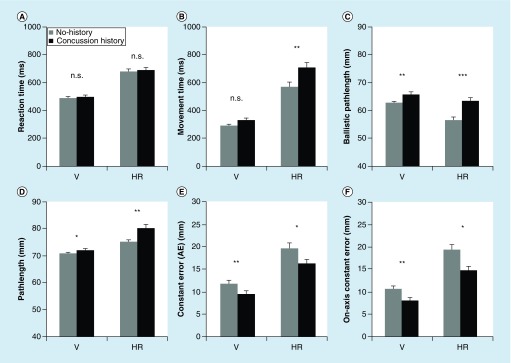
**Behavioral performance as a function of task complexity and concussion history.** Movement timing values of **(A)** mean reaction time and **(B**) mean movement time, for both groups (concussion history, no history) across both conditions (V, HR). Movement execution values of **(C)** mean ballistic pathlength, **(D)** mean full pathlength, **(E)** mean AE (constant error) and **(F)** mean absolute on-axis values for both groups (concussion history, no history) across both conditions (V, HR). *p < 0.05, **p < 0.01. ***p < 0.001. Error bars represent standard error of the mean. AE: Constant absolute error, end point accuracy; HR: Horizontal rotated; V: Vertical.

Repeated-measures mixed ANOVA revealed a significant main effect of condition on RT (RT was significantly longer in HR than in V), but no main effect of group or group × condition interaction. Thus, there was no RT difference between concussion history and no history control participants in V and HR, as confirmed by pair-wise analysis (compare Figure 2A). In contrast, ANOVA yielded a significant main effect of group, condition and group × condition interaction on MT. MT was longer in HR than in V, was longer for participants with concussion history than for no history healthy controls across conditions, and the significant interaction between group × condition revealed that this effect was even more pronounced in HR than in V for the concussed group (compare Figure 2B). For TMT, CMT and PeakVel, repeated-measures mixed ANOVA revealed significant effects for condition, but not for group and group × condition. TMT, CMT and PeakVel were significantly longer in HR than in V, independent of group.

### Pathlength

Repeated-measures mixed ANOVA yielded a significant main effect of group, condition and group × condition interaction on ballistic pathlength. BallPL was longer for participants with concussion history compared with healthy controls, independent of condition HR and V, and was more pronounced in HR compared with V, independent of group. The interaction group × condition showed that healthy control participants without concussion history had a smaller BallPL (i.e., were able to correct the initial movement path quicker) compared with participants with concussion history, and that this effect was more pronounced in the CMI condition (HR). This result was confirmed by pair-wise comparisons showing a significant difference between concussion history and healthy control in HR and V (compare Figure 2C). Also, ANOVA revealed significant effects for group, condition and group × condition for FullPL. FullPL was significantly longer in concussion history compared with healthy control participants, independent of condition HR and V, and was significantly longer in HR compared with V for participants with concussion history compared with controls. Pair-wise comparison showed that there was a significant difference between concussion history and control in V and HR (compare Figure 2D). For CorrPL, ANOVA showed significant effects for condition, but not for group and group × condition. CorrPL was shorter in V than in HR, independent of group. In accordance to that, pair-wise comparison showed no significant CorrPL difference between concussion history and control participants in condition HR and in V.

### End point analysis

Repeated-measures mixed ANOVA revealed significant main effects for AE and AbsOn (i.e., distance error) for group and condition, but not a group × condition interaction. AE and AbsOn were smaller in concussion history compared with healthy control participants, and were higher in the HR condition compared with V, independent of group. Pair-wise comparison showed for both variables a significant difference between concussion history and control participants in HR and V (compare Figure 2E & F). For AbsOff (i.e., direction error), ANOVA yielded significant main effect for condition, but not for group and group × condition. AbsOff was higher in HR than in VR, independent of group. ANOVA for VE yielded no significant effects for condition, group and group × condition.

### Direction reversals

One-way ANOVA for DR revealed no significant effects for group. The number of DR was similar for control and concussion history participants. We also did not find any significant differences in the remaining error counts described in the method section between both groups (all p > 0.05).

### Performance as a level of dissociation

**Figure 3.**
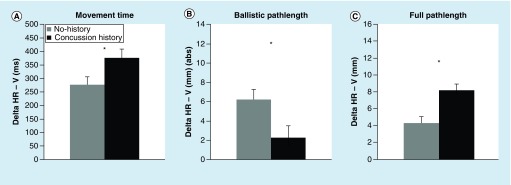
**Movement timing and movement execution as a function of dissociation from direct interaction (i.e., horizontal rotated values–vertical values).** **(A)** Mean movement time values for the nonstandard mapping condition (HR) subtracted from the standard mapping condition (V) for both groups (concussion history, no history). **(B)** Mean ballistic pathlength absolute values for the nonstandard mapping condition (HR) subtracted from the standard mapping condition (V) for both groups (concussion history, no history). **(C)** Mean full pathlength absolute values for the nonstandard mapping condition (HR) subtracted from the standard mapping condition (V) for both groups (concussion history, no history). HR: Horizontal rotated; V: Vertical.

Statistical outcomes of the one-way ANOVA for the level of dissociation are summarized in Table 4, and descriptive statistics for all dependent variables and both groups (concussion history, no history) are summarized in Table 5. ANOVA's yielded significant effects of group (concussion history, no history) for MT, BallPL and FullPL, all other variables showed no significant differences. δHR-V of MT was higher, δHR-V for BallPL was shorter and δHR-V for FullPL was larger in participants with concussion history compared with the healthy control group (compare Figure 3A–C).

**Table 4.** **Statistical outcome of the one-way ANOVA for the level of dissociation (i.e., dependent variable horizontal rotated subtracted from vertical) analysis for all dependent variables with the between factor group (concussion history, no history).**

**Variable**	**Group**
RT	F(1,98) = 0.00^†^

MT	F(1,98) = 4.93*

TMT	F(1,98) = 1.46^†.^

CMT	F(1,98) = 0.18^†^

PeakVel	F(1,98) = 0.98^†^

BallPL	F(1,98) = 6.04*

FullPL	F(1,98) = 6.20*

CorrPL	F(1,98) = 0.00^†^

AE	F(1,98) = 0.59^†^

VE	F(1,98) = 2.50^†^

AbsOn	F(1,98) = 1.89^†^

AbsOff	F(1,98) = 0.02^†^

^†^Non-significant values.

*p < 0.05.

AE: Constant absolute error, end point accuracy; AbsOff: Absolute off-axis error; AbsOn: Absolute on-axis error; BallPL: Ballistic pathlength; CMT: Corrective movement time; CorrPL: Corrective pathlength; FullPL: Full pathlength; MT: Movement time; PeakVel: Peak velocity; RT: Reaction time; TMT: Total movement time; VE: Variable error, end point precision.

**Table 5.** **Descriptive statistics of the one-way ANOVA for level of disassociation.**

**Variable**	**Concussion history (mean ± SD)**	**No history (mean ± SD)**
**ms**		

RT	191.95 ± 123.70	191.36 ± 126.40

MT	376.79 ± 240.33	277.50 ± 202.52

TMT	680.47 ± 331.73	598.31 ± 345.61

CMT	303.68 ± 205.05	320.82 ± 196.98

PeakVel	-83.99 ± 40.85	-76.28 ± 36.35

**mm**		

BallPL	-2.27 ± 8.42	-6.23 ± 7.57

FullPL	8.22 ± 9.74	4.33 ± 5.02

CorrPL	10.49 ± 8.64	10.56 ± 8.26

AE	6.78 ± 6.44	7.85 ± 7.47

VE	1.07 ± 4.64	-0.34 ± 4.26

AbsOn	6.70 ± 6.37	8.69 ± 7.97

AbsOff	2.56 ± 3.21	3.21 ± 2.29

AbsOff: Absolute off-axis error; AbsOn: Absolute on-axis error; AE: Constant error, end point accuracy; BallPL: Ballistic pathlength; CMT: Corrective movement time; CorrPL: Corrective pathlength; FullPL: Full pathlength; MT: Movement time; PeakVel: Peak velocity; RT: Reaction time; SD: Standard deviation; TMT: Total movement time; VE: Variable error, end point precision.

### Relationship between performance deficit & concussion history

**Figure 4.**
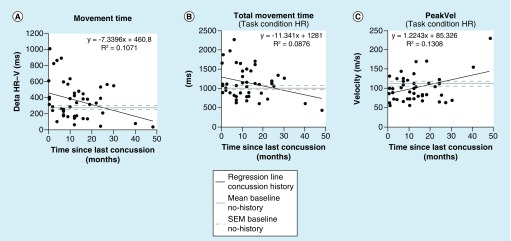
**Correlation of performance score and concussion history time (i.e., the time point of the last concussion) for participants with concussion history.** The graph shows the relationship between **(A)** MT values for the nonstandard mapping condition (HR) subtracted from the standard mapping condition (V) and the time since the last concussion for participants with concussion history, plotted as linear function. The graph also shows the mean MT values for the nonstandard mapping condition (HR) subtracted from the standard mapping condition (V) of control participants with no history of concussion (i.e., the baseline performance of healthy age-matched participants) plotted as grey solid line along the x axis, and baseline SEM represented by the both grey dashed lines along the x axis. **(B & C)** show similar information, but for the relationship between TMT **(B)** and PeakVel **(C)** of HR and the time since the last concussion for the participants with concussion history. Note that in all significant correlations and graphs, respectively, the regression line of the performance of participants with concussion history crossed the baseline performance of participants with no history of concussion at approximately 24 months. HR: Horizontal rotated; MT: Movement time; SEM: Standard error of the mean; V: Vertical.

A correlation analysis yielded a significant relationship between some of our dependent variables and the time since concussion in this age group. We observed significant relationship between time since concussion and MT δ (HR-V, r = 0.327; p < 0.05), MT in the HR condition (r = 0.332; p < 0.05), TMT in the HR condition (r = 0.296; p < 0.05) and PeakVel in the HR condition (r = 0.362; p < 0.05). All other dependent variables showed no significant relationship to time since last concussion (all p > 0.05). Figure 4A shows the fitted regression line of the relationship between the performance variable MT δ (the MT increase for the CMI condition) and the time since the last concussion. Also shown is the mean MT δ of the participants with no history of concussion (along the x axis) as baseline performance healthy controls. Notably, the regression line for participants with a concussion history does not cross the baseline of the control participants until approximately 24 months. A similar pattern was observed for the other significant variables that showed significant regressions to time since last concussion, as for example presented in (Figure 4B) (TMT in condition HR) and (Figure 4C) (PeakVel in condition HR).

### Discriminant analysis

**Figure 5.**
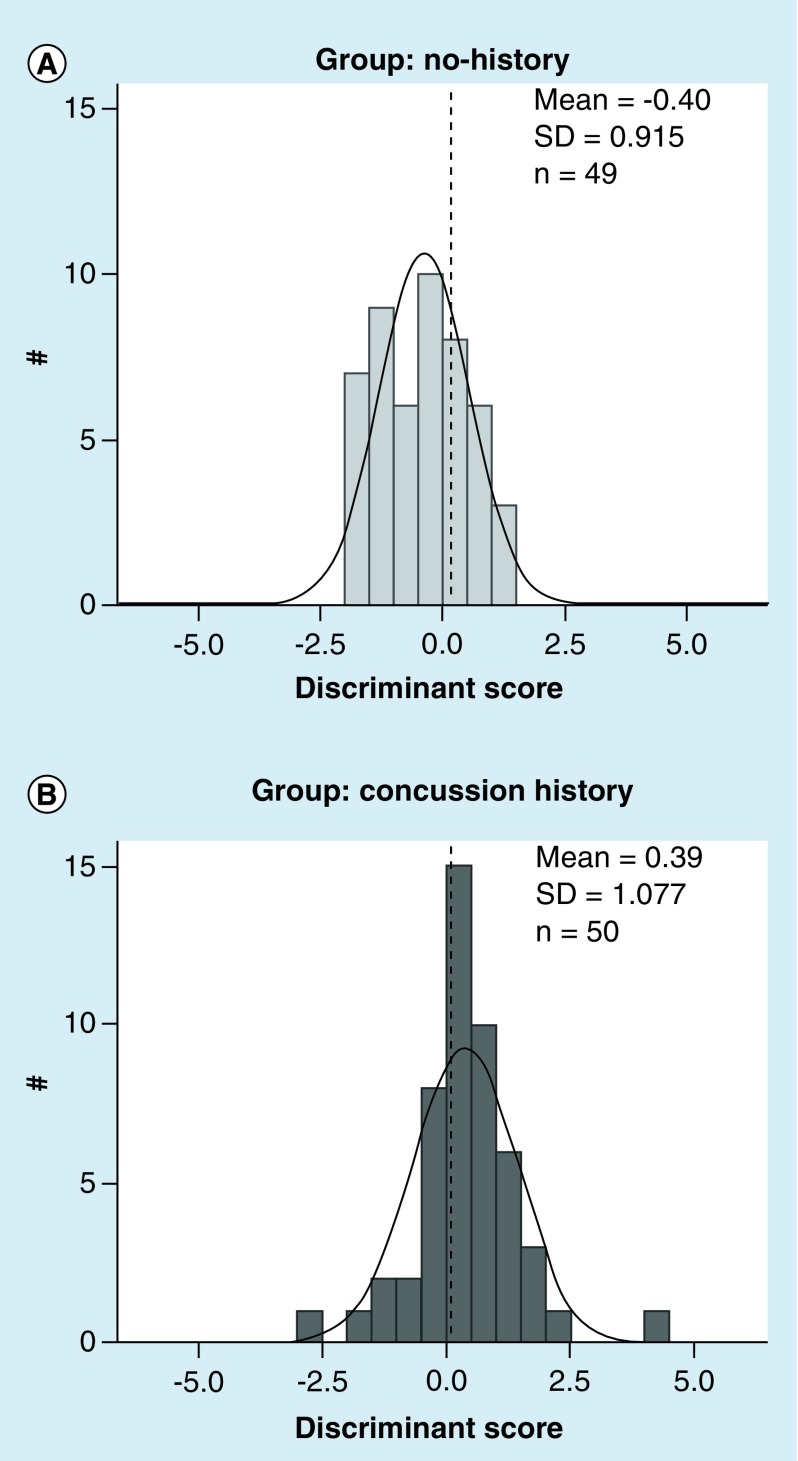
**Step-wise discriminant analysis results.** Normal distribution of discriminant scores for participants **(A)** from the control group with no history of concussion and **(B)** with concussion history. Scores are a weighted contribution of dependent variables chosen by the analysis to maximize differentiation between groups. Note the difference between participant groups. SD: Standard deviation.

The discriminant analysis demonstrated a good separation of participants with concussion history from the no-history control group. Based on the outcome of the results presented above, the predictors we entered in the discriminant function classifying concussion history versus no history participants were the dependent variables that reached significance in the main repeated measures-mixed ANOVA, that is, MT, BallPL, FullPL, AE, AbsOn of HR condition, and BallPL, FullPL, CorrPL, AE, AbsOn of V, and MT, BallPL, FullPL of δHR-V. The outcome of the discriminant analyses showed that our assessment tool was able to discriminate participants with a history of concussion from healthy age-matched controls with no history of concussion with an accuracy of 70% (for details please see Table 6 & Figure 5).

**Table 6.** **Classification results^†,‡^ of the step-wise discriminant analyses.**

**Analysis**	**Group**	**Predicted group membership**		**Total**

		**Concussion history**	**No history**	
Original:				
– Count	Conc. Hi.	37	13	50
	No Hi.	17	32	49
– %	Conc. Hi.	74	26	100
	No Hi.	34.7	65.3	100

Classification^§^:				
– Count	Conc. Hi.	36	14	50
	No Hi.	17	32	49
– %	Conc. Hi.	72	28	100
	No Hi.	34.7	65.3	100

^†^69.7% of original grouped cases correctly classified.

^‡^68.7% of cross-validated grouped cases correctly classified.

^§^Cross validation is done only for those cases in the analysis. In cross validation, each case is classified by the functions derived from all cases other than that case.

Conc. Hi.: Concussion history; No Hi.: No history.

### Discussion

The present study sought to determine whether asymptomatic children and adolescents with a history of concussion displayed visuomotor impairments while performing CMI tasks. The results clearly demonstrate performance differences on complex visuomotor tasks between children and adolescents with a history of concussion and healthy aged-matched controls with no history of concussion. In accordance with our hypothesis, we found prolonged visuomotor deficits in children and adolescents with a history of concussion. As in our previous study with adult athletes with a history of concussion [22], the visuomotor deficits were most pronounced when there was dissociation between vision and action, and were observed for both movement planning and movement execution. Notably, movement execution performance deficits in children and adolescents with a history of concussion were exacerbated compared with the previous study. Furthermore, we found a significant relationship between impaired performance variables and the time since last concussion: deteriorated movement timing variables of children and adolescents with concussion history matched the performance of healthy age-matched controls with no history of concussion only after approximately 24 months.

The results of the current study demonstrate that children and adolescents with a history of concussion have, despite being asymptomatic and back at play, deficits with a rule-based movement task. These findings complement our previous studies on adult athletes with concussion history, as well as our studies with adults at-risk for the development of AD (through an MCI diagnosis or family history [23,24]). While similar behavioral deficits between these very different groups do not mean that the underlying mechanism is the same, it suggest that children, adolescents and young adults with a history of concussion are neurologically fragile even when they are classified as being asymptomatic. Our task that ‘pushes the system’ appears to bring out behavioral deficits across a range of mild brain dysfunction, even, as shown for the concussion history participants, when asymptomatic and through their return to play schedule.

We postulate that these deficits may be due to disruptions in fronto-parietal networks and the communication between brain regions that are responsible for planning and executing skilled movement when there is an element of cognition involved. This conclusion is based on recent work on brain networks for CMI in both human and nonhuman primates [26,28,30,31,41,42]. In particular, the network alterations may arise from impairments in communication between cortical and subcortical movement control regions due to cellular damages following concussion. Indeed, several other current studies provide evidence for network changes in the human brain following concussion, showing cellular changes as reduced cerebral blood flow and cerebral glucose utilization, all changes that could lead to ATP metabolism and long-term potentiation problems [43–50]. It is becoming increasingly clear that oxidative stress may trigger neuropathological processes both local and distal in addition to mild brain contusion, even when they may only manifest themselves months to years later [50]. Recent brain metabolism studies in adults with concussion history have found concurrent motor skill deficits and primary motor cortex metabolism abnormalities related to deficits in motor learning [51].

In accordance with our proposal, other colleagues have found alterations in both function and anatomy, in particular, brain regions following concussion using imaging and brain function studies, reporting increased and more widespread activation of prefrontal cortex, dorsolateral prefrontal cortex and cerebellum for a variety of tasks in concussed versus nonconcussed populations [15,52,53]. The additional network activations were suggested to be due to compensatory mechanisms to accommodate functional and/or structural deficits in the brain's default networks as a result of concussion (see above). In particular, for networks that may be specifically related to CMI, studies with concussed individuals have found an increased activation in parietal, frontal, as well as cerebellar regions when compared with preinjury fMRI data, although no cognitive decline was noted [27]. Indeed, recent anatomical studies have found altered diffusion characteristics within white matter tracts in concussed adolescents, younger and older adults with concussion history exactly in those pathways connecting frontal and parietal regions [29,54–56]. Again, these data are in line with our group's findings on the crucial role played by such fronto-parietal networks in integrating thought and action [26,28,32,57].

In support of our second hypothesis, we observed not only similar but also additional performance deficits for children and adolescents with concussion history compared with young adults with concussion history (data from a previous study [22]). In both studies, concussion history participants demonstrated impaired movement timing (as MT). However, in the present study, participants with concussion history showed additional deficits affecting movement execution (as BallPL and FullPL). Although we were not able to combine and directly compare these datasets (for technical reasons), the overall findings from both studies may reflect the fact that children and adolescents are not only more vulnerable to receiving a concussion [7], but that the growing brain of children and adolescents is also neurologically more fragile for executing CMI tasks than adults following a concussion. This conclusion supports previous work showing that the adolescent brain may differ from the adult brain also following a concussion [58,59], specifically for frontal versus parietal neuronal systems [40].

Similar to our previous study [22], the prolonged ballistic MT (i.e., the MT until participants were able to make their first initial movement path correction) in the cognitively challenging movement condition was again the most prominently affected performance measure. This parameter was also one that showed a significant relationship with the time since last concussion in the present study. We further observed three other movement parameters that changed significantly with the time since concussion, reaching the performance of those children and adolescents with no concussion history after only 2 years (compare Figure 4A–C). We recognize the fact that the two participants having the longest time pass since their most recent concussion showed the strongest performance, and that we have fewer participants with that long a time since their last concussion in our dataset. Future research will involve more participants with concussion history between 30 and 50 months postconcussion, to fill that gap. Nevertheless, the mean time postconcussion was 13 months in our present dataset. Thus, we present robust evidence for prolonged performance deficits of children and adolescents with a history of concussion compared with age-matched participants with no history of concussion.

Indeed, another study with adults having concussion history (who had been cleared to return to play after 10 days) showed that even after 12 months, brain cortical activity did not return to preinjury status [14]. In this study, no follow-up measures were taken after 12 months, so we cannot directly compare the recovery timelines of these two age groups. However, our findings also align with observations of brain activity alterations in the adult brain after 3 years postconcussion, in particular for force production tasks and balance tests [60,61]. These results and our present findings underline that the human brain seems to be affected much longer following a concussion than a few weeks even if classified as being asymptomatic, after pursuing all steps of current return to play protocols and being back to full team sport activity. Importantly, our results might also explain the higher risk for a reinjury [2,3]. If the CMI brain networks needed for integrating vision, thought and action are not working and communicating properly, one would move, react and correct more slowly on the field relative to nonconcussed peers, increasing one's vulnerability to reinjury. We are currently examining this performance recovery on an individual longitudinal basis (versus the cross-sectional approach used here) to more comprehensively characterize recovery of functionally relevant skill following head injury.

In the present study, the participants with concussion history executed the tasks more slowly, but were also more accurate at reaching the center of the target at the end of their movement. This interesting observation might reflect a speed–accuracy trade off [61,62], maybe because they executed the task more cautiously due to their history of a traumatic brain injury [63]. Note that we instructed the participants to slide the finger as quickly and accurately as possible to the target. Furthermore, we observed that the performance deficits were not based solely on a speed-accuracy trade off, since participants with concussion history also had a longer path length. Thus, we suggest our task is revealing further disturbances in the motor and/or CMI networks. We note however, that it would be interesting to compare whether the speed in the few training trials was the same between the concussion history participants and the no-history controls, that is, whether or not both groups differed already in their motor execution. As mentioned above, this was beyond the scope of the current cross-sectional study, and is more appropriate for a longitudinal study (ongoing).

Our discriminant function analysis tested the predictive power of our task to differentiate children and adolescents with and without concussion history. Here, our analysis was less effective (70%; compare Table 6 & Figure 5) compared with our previous study with young adult athletes with history of concussion (94%) and early AD patients (86%) [22,26]. We suggest two possible explanations for this lower result: first, one might argue that by reducing our setup to only two conditions (V, HR) rather than the four to seven conditions from our other studies (due to time constraints), we likely lost data power to assess all relevant CMI functions. A second possibility may relate to behavioral differences in the children's and adolescents’ growing brains, since we had a range between 9 and 16 years of age in our study, a time period that is well known for sex- and age-related differences compared with young adults [7,40]. However, we found no statistical age- and sex-related behavioral differences in our current data analysis. With the current study, we were unfortunately unable to separate the data pool into an adequate balanced group of female and male subjects for each age group that are well known to show age- but also sex-related performance differences, as for example, 8–10 years, 11–13 years and 14–16 years [40]. However, age- and sex-related differences in the performance of children and adolescents following concussion were not the main aim of the present study, but should be further assessed in future studies when larger datasets are available.

To summarize, we observed prolonged CMI deficits in asymptomatic children and adolescents with concussion history that notably were all back to their full-sport activity. This finding suggests that current return to sport/school/work assessments that do not test CMI, crucial for skilled activities, may not fully capture functional abilities postconcussion. Automated, objective testing for concussion is a hot topic; determination of the presence and degree of a concussion can be difficult and time consuming [13]. Our experimental task is one of the first that combines testing the elements of cognition and motor action simultaneously, and identifies CMI deficits where other testing procedures do not. Thus, it appears that our task may be helpful as a feasible, cheap and quick assessment tool for in field measurements or in clinics to analyze the functional recovery of those affected by concussion.

## Conclusion

Asymptomatic children and adolescents with a history of concussion showed a distinct performance decline for a prolonged period of time (2 years) following their last concussion, a finding which is highly important for future discussion about current return to play guidelines and reducing the risk of reinjury in this age group. We suggest that examining performance requiring the integration of cognition and action is a useful and important approach to comprehensively assess brain function following concussion.

**Executive summary**

- We investigated whether asymptomatic children and adolescents with a history of concussion show prolonged cognitive–motor integration (CMI) deficits.
- In total, 50 asymptomatic children and adolescents with concussion history and 49 controls with no history performed two tasks on a dual-touchscreen laptop.
- In the one task, target location and motor action aligned, and in the CMI task, target location and motor action decoupled.
- We observed significant CMI impairments in children and adolescents with concussion history.
- We also observed a correlation between time since last concussion and CMI deficit; participants with concussion history did not match the baseline level of no history controls until nearly 2 years post concussion.
- Based on previous findings, we suggest that these performance deficits are due to concussion-induced disruptions in the fronto-parietal networks responsible for rule-based movement guidance.
- The observed prolonged CMI deficits suggest that current return to sport/school/work assessments that do not test CMI, crucial for skilled activities, are not fully capturing functional abilities post concussion.
